# Palliative care for patients with cancer: do patients receive the care they consider important? A survey study

**DOI:** 10.1186/s12904-018-0315-3

**Published:** 2018-04-17

**Authors:** Marianne Heins, Jolien Hofstede, Mieke Rijken, Joke Korevaar, Gé Donker, Anneke Francke

**Affiliations:** 1 Netherlands Institute for Health Services Research (NIVEL), P.O Box 1568, 3500 BN Utrecht, The Netherlands; 2 Amsterdam Public Health research institute, VU University Medical Center, Amsterdam, the Netherlands; 3 Expertise center palliative care, VU University Medical Center, Amsterdam, the Netherlands

**Keywords:** Palliative care, Primary health care, Home care services, Neoplasms, Quality of care

## Abstract

**Background:**

In many countries, GPs and home care nurses are involved in care for patients with advanced cancer. Given the varied and complex needs of these patients, providing satisfactory care is a major challenge for them. We therefore aimed to study which aspects of care patients, GPs and home care nurses consider important and whether patients receive these aspects.

**Methods:**

Seventy-two Dutch patients with advanced cancer, 87 GPs and 26 home care nurses rated the importance of support when experiencing symptoms, respect for patients’ autonomy and information provision. Patients also rated whether they received these aspects. Questionnaires were based on the CQ index palliative care.

**Results:**

Almost all patients rated information provision and respect for their autonomy as important. The majority also rated support when suffering from specific symptoms as important, especially support when in pain. In general, patients received the care they considered important. However, 49% of those who considered it important to receive support when suffering from fatigue and 23% of those who wanted to receive information on the expected course of their illness did not receive this or only did so sometimes.

**Conclusion:**

For most patients with advanced cancer, the palliative care that they receive matches what they consider important. Support for patients experiencing fatigue may need more attention. When symptoms are difficult to control, GPs and nurses may still provide emotional support and practical advice. Furthermore, we recommend that GPs discuss patients’ need for information about the expected course of their illness.

## Background

Although survival in patients with cancer has increased markedly in the past few decades, more than a third of patients with cancer still die within 5 years of the diagnosis [1–4]. This is often preceded by a period of months, sometimes years, in which patients receive palliative care. When cure or life prolongation is the main goal, care for patients with cancer is mostly provided by hospital professionals. At the end of life, when the main goal of care is improvement in the quality of life rather than cure or life prolongation, most patients prefer to remain at home [5]. In countries with a strong primary care system, general practitioners (GPs) and home care nurses are often closely involved in the care for patients with advanced cancer [6–12]. The involvement of GPs and home care nurses has several benefits: patients are more likely to die in their preferred place [13, 14] and they are less likely to have emergency department visits [15]. At the same time, the care needs of patients with advanced cancer are complex. Patients often suffer from a combination of complex symptoms, such as pain, fatigue, dyspnoea, anxiety and depression [16]. Given the varied and complex needs of these patients, providing satisfactory care and support in managing these symptoms is a major challenge for the GPs and home care professionals involved.

Besides providing support when a patient suffers from physical or psychological symptoms, GPs and home care professionals may also find it challenging to ensure good information provision and respect for the patient’s autonomy. Patients’ preferences are patient-specific and may change over time, and patients may eventually experience problems expressing their wishes. The medical and scientific community is increasingly aware of the importance of respecting patients’ autonomy and of satisfactory information provision. This is implemented through the concept of advance care planning: the process of discussing in good time and recording patient preferences concerning the goals of palliative care, and of subsequently planning this care [17]. Advance care planning poses challenges for healthcare providers, such as GPs and home care nurses, in ensuring proper information provision and respecting the autonomy of patients [17].

In addition, studies of the quality of palliative care indicate that symptoms are not always managed properly [18–22], and that information provision and respecting the patient’s autonomy could be improved [21, 22]. However, in these studies information was often collected from relatives rather than directly from patients themselves, while other studies did not focus specifically on patients with advanced cancer. Given this lack of knowledge about the specific preferences and experiences of patients with advanced cancer, more insight is needed into their opinions about these subjects in order to optimise these aspects of palliative care. Also, given the important role of GPs and home care nurses in palliative care, it could be helpful to have insight into the extent to which their perspective on respecting autonomy and information provision match with that of their patients with advanced cancer. We therefore formulated the following research questions for this study:

1. What aspects of care do patients with advanced cancer consider important regarding support when experiencing specific symptoms, respect for autonomy and the provision of information?
2. Do the perspectives of patients regarding respect for autonomy and information provision match with what GPs and home care nurses consider important?
3. Does the care that advanced cancer patients actually receive match with what they consider important regarding care for physical and psychosocial well-being, respect for autonomy, and information?

## Methods

### Study design

We applied a stepwise approach. First we recruited GPs, who then selected patients with advanced cancer in their practice according to the selection criteria provided by the researchers and invited them to participate in the study. Patients who gave informed consent to participate in the study and were receiving home care were asked to invite one of their home care nurses (registered nurses or certified nurse assistants) to participate.

#### Participants

We randomly selected 3000 GPs from the national GP registry, which holds the addresses of all the GPs in the Netherlands. They were sent an information letter and reply form. In the information letter they were asked whether they were willing to recruit one or more of their advanced cancer patients and – if so – whether they were also willing to complete a questionnaire about these patients. GPs who replied that they were willing to participate were sent a written questionnaire for themselves or, if they preferred, a link to the online version of the questionnaire. Besides, these GPs were sent information letters and questionnaires on paper for their patients and home care nurses. The GPs were asked to recruit patients and hand the information letters and questionnaires to patients with a diagnosis of cancer with a poor prognosis (i.e. the GP would not be surprised if the patient were to die within a year). Patients in the terminal phase or those who were mentally or cognitively unable to fill in a questionnaire in Dutch had to be excluded. There was no restriction on the number of patients that GPs could recruit.

In the information package that the patient received from the GP (which included an information letter and questionnaires), the patient was also asked to pass an information letter and questionnaire on to one of their home care nursing staff if they were receiving home care.

### Measures

For data collection among patients, we used the following sections from the Consumer Quality Index (‘CQ Index’) Palliative care [21]: ‘Care for physical and psychosocial well-being (with items on support when a patient had specific symptoms)’ ‘Respect for autonomy’ and ‘Information’.

The CQ Index Palliative Care is a valid and reliable instrument [23] that contains questions about how important certain care aspects are for patients or relatives (importance items) and corresponding questions about actual care experiences (experience items). For the importance items, patients are asked to rate the importance of items on a four-point Likert-scale, ranging from ‘not important’ to ‘very important’. For the experience items, patients are asked to rate their experience on a four-point Likert-scale, ranging from ‘no/never’ to ‘always’. For the questions about support when experiencing specific symptoms, the option ‘not applicable’ could be chosen if a patient had not experienced a symptom. The original CQ Index Palliative Care asks about care given by care professionals in general, while we asked specifically about care received from a GP or home care nurse.

In the questionnaires for the GPs and home care nurses, we rephrased the questions to reflect the perspective of the GP or home care nurse. For instance, ‘Are you involved in decisions about your care?’ (which was an item in the section on ‘Information provision’) was changed to ‘Do you involve patients in decisions about their care?’ Not all of the questions we asked patients were applicable to GPs and home care nurses. For instance, we did not ask the GPs and nurses whether they gave patients contradictory information or a clear explanation, as we expect that GPs and home care nurses will try to give consistent and understandable information. We also did not include questions about support when a patient suffers from a specific symptom, as patients may receive this support from another health professional.

### Statistical analyses

Descriptive statistics - frequencies and percentages or means (with 95% confidence intervals), depending on the type of variable - were computed for the background characteristics and importance scores. In the analyses of the experience scores, we excluded patients who rated an aspect as ‘not important’. Analyses were performed using Stata/SE 14.2.

## Results

### Participants

A total of 87 GPs participated in the study. They were predominantly male (57%) and had on average 19 years of experience as a GP. 60% had received some form of palliative care education, mostly special courses.

Between June 2015 and January 2016, we recruited 72 patients through the participating GPs. Half of the patients were male and their mean age was 70. Most were diagnosed with lung or colorectal cancer. The time since diagnosis ranged from less than 6 months to over 5 years (Table 1).

**Table 1:** Background characteristics of patients

Background characteristic	Patients (*n* = 72)
Age (yrs.)	70.5 (8.9)
Sex	
Male (%)	35 (49%)
Female (%)	37 (51%)
Cancer site	
Lung	21 (29%)
Colorectal	15 (21%)
Breast	11 (15%)
Prostate	6 (8%)
Pancreatic	4 (5%)
Time after diagnosis	
< 6 months	18 (25%)
6 months – 1 year	13 (18%)
1–2 years	16 (22%)
2–5 years	19 (26%)
> 5 years	6 (8%)
Home care	
No	32 (44%)
Personal care	30 (42%)
Medical care	20 (28%)
Housekeeping	14 (19%)
Missing	1 (1%)

We recruited 26 home care nurses via the participating patients. The nurses were predominantly female (85%). They had on average 11 years of experience as a home care nurse and almost all (88%) had received some form of palliative care education.

### What aspects of care do patients consider important?

**Fig. 1:**
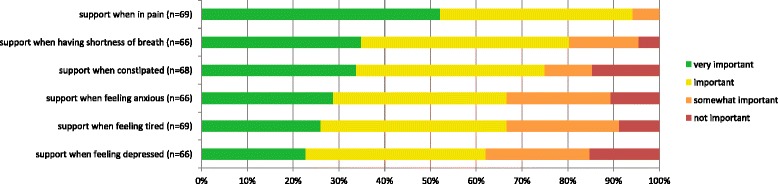
Perceived importance of aspects of support when having a specific symptom as rated by patients

The majority of the patients considered support when experiencing specific symptoms as important or very important, although some patients rated items as not important (9–15%) (Fig. 1). Mean importance scores were between 2.7 and 2.9 on a four-point scale (Table 2). An exception was ‘support when in pain’ and ‘support when experiencing shortness of breath’, which hardly any patients rated as not important (0 and 4%, respectively) (Fig. 1).

**Table 2:** Mean importance score (95% CI) for aspects of respect for autonomy and information provision as rated by patients, GPs and home care nurses

Item	Patients	GPs	Home care
Care for physical and psychosocial well-being			
Support when in pain	3.46 (3.32–3.61)	–	–
Support when experiencing shortness of breath	3.11 (2.90–3.31)	–	–
Support when constipated	2.94 (2.69–3.19)	–	–
Support when feeling anxious	2.85 (2.61–3.09)	–	–
Support when feeling tired	2.84 (2.62–3.06)	–	–
Support when feeling depressed	2.70 (2.45–2.94)	–	–
Respect for autonomy			
Patient is involved in decisions about care	3.63 (3.50–3.75)	3.87 (3.78–3.95)	3.96 (3.88–4.04)
Professional caregivers take personal preferences into account	3.58 (3.45–3.71)	–	–
Information provision			
Professional caregivers give consistent information	3.61 (3.48–3.74)	–	–
Patient knows who the contact person is for care	3.56 (3.41–3.70)	3.66 (3.55–3.77)	3.88 (3.75–4.02)
Patient receives information about benefits and risks of treatment	3.51 (3.34–3.69)	3.63 (3.52–3.74)	–
Professional caregivers explain things in a way patient understands	3.51 (3.37–3.66)	–	–
Patient receives information about expected course of the illness	3.39 (3.19–3.58)	3.61 (3.49–3.73)	–

**Fig. 2:**
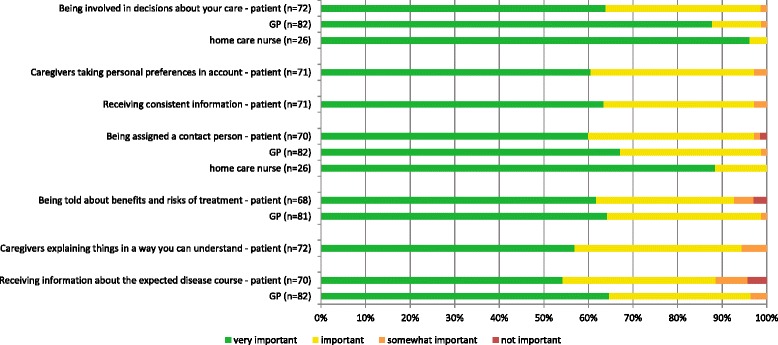
Importance of aspects of respect for autonomy and information provision as rated by patients, GPs and home care nurses

Almost all patients rated items related to ‘respect for autonomy’ or ‘information provision’ as important or very important, ranging from 89% for receiving information about the expected course of the disease to 99% for being involved in decisions about care (Fig. 2). Mean importance scores ranged from 3.4 to 3.6 on a scale of 1 (not important) to 4 (very important).

### What do GPs and home care nurses consider important, and does this match with patients’ opinions?

Almost all GPs rated aspects of ‘respect for autonomy’ and ‘information provision’ as important or very important (96–98%). They considered these aspects even more important than patients themselves (Table 2). Almost all home care nurses considered the items ‘involving patients in decisions’ and ‘assigning a contact person’ as very important (96 and 88%, respectively). None of the GPs or home care nurses rated an aspect as not important (Fig. 2).

### Do patients receive the care they consider important?

**Fig. 3:**
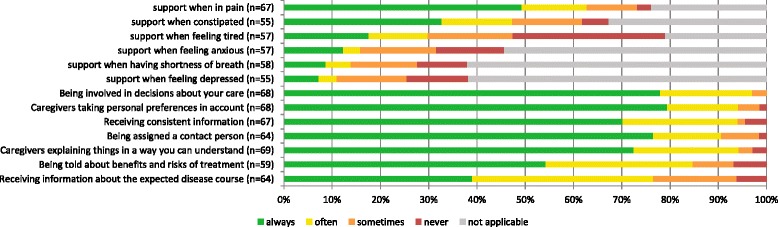
Received aspects of care rated by patients who find these aspects important*. * patients who rated an aspect as not important were not included

Some patients who considered receiving support when experiencing a certain symptom to be at least somewhat important did not receive this support or only did so sometimes. For most symptoms the proportion ranged between 20 and 30%. The exceptions were feeling tired, for which almost 50% of the patients did not receive support or only received support sometimes, and being in pain, for which only 13% received no or only occasional support (Fig. 3).

As to respect for autonomy and information provision, most patients indicated that they did indeed receive the care they considered important. The only aspect that a considerable number of patients considered important but did not receive or only received sometimes was information about the expected course of the illness (23% of those who considered it at least somewhat important to receive this information) (Fig. 3).

## Discussion

### Main findings

Patients with advanced cancer as well as their GPs and home care nurses considered respect for autonomy and information provision to be very important and most patients did indeed receive these aspects of care. Patients considered support when experiencing specific symptoms somewhat less important. However, about a third of those who considered it important to receive support from the GP or home care nurse when they experienced a specific symptom indicated that they did not receive this support or only did so sometimes. For fatigue, this was the case for almost half of the patients. An exception was support when in pain, which patients considered more important than support for other symptoms and which they also received more often.

### Strengths and limitations

A strength of our study is that we asked patients, their GPs and their home care nurses about their opinions and experiences regarding important aspects of palliative care. This enabled us to compare different points of view. We chose a stepwise inclusion process, first inviting GPs, who then asked patients, who then asked nurses. The advantage was that we could make couples of patients and GPs and GPs were familiar with the situation of the patients and could therefore assess whether patients were able to be contacted and interviewed. However, the stepwise inclusion process may have led to selection bias. Firstly, few GPs responded to the study invitation, the response rate was very low (3%), so we may for example have selected GPs who were specifically interested in the care for advanced cancer patients. Nevertheless, the age and sex profiles of the participating GPs were quite similar to the nationwide figures (mean age of 51 vs. 48 and 42% vs. 48% female) [24]. Secondly, GPs may have selected patients with whom they had a good relationship and patients may have done the same in selecting home care nurses. Our results could therefore be too positive. Thirdly, patients may have felt obliged to respond positively when answering questions about their care that their GP had asked them to answer. However, as patients filled in the questionnaires at home and sent them to the researchers, we think it is not very likely that this influenced the results. An alternative method could have been for instance, to select patients through internet fora, which possibly would have led to lower appreciation for the care received, as it is conceivable that patients with negative experiences are more active on these fora. Another alternative inclusion method could have been via the treating specialist, yet, this route would have exclude patients who are no longer being treated by their oncologist, resulting in bias by disease stage.

Another limitation related to our stepwise recruitment is that the number of home care nurses participating in the study was relatively low (*n* = 26). This is because only a limited number of patients were receiving home care delivered by nurses (*n* = 32). As we received questionnaires from most of these nurses, we can assume no selection took place of nurses who were particularly interested in palliative care or had a good relationship with the patient. A validation study using a different sampling method would be interesting.

### Interpretation of the study results in relation to existing literature

A comparison of our results with an earlier study of patients’ opinions and experiences of palliative care for patients with varying diseases (cancer, chronic obstructive pulmonary disease (COPD), heart failure and other non-cancer diseases) reveals some differences [21]. The patients with advanced cancer in our study considered respect for autonomy as more important than a broader group of patients receiving palliative care (3.58–3.63 vs. 3.46–3.49). Patients in our study considered aspects of support when experiencing specific symptoms to be less important (2.70–3.11 vs. 3.04–3.36), with the exception of support when in pain, which both samples considered equally important (3.46 vs. 3.51). This may be explained by findings of previous studies that cancer patients have more palliative care, palliative treatments, discussion of end-of-life topics and advance care planning than palliative patients without cancer [6, 25]. Cancer patients may thus have fewer unmet needs as to support from professionals when experiencing symptoms.

In line with the results of our study, previous studies found that most cancer patients had talked to their GPs about life expectancy, possible medical complications and the burden imposed by treatments [6, 25]. GPs are generally better able to foresee imminent death in patients with cancer compared with patients with COPD or chronic heart conditions [26]. This is related to the fact that the disease trajectory is often less variable in cancer than in other chronic life-threatening disease [27], which may facilitate discussing prognosis [28]. This may explain why GPs are more likely to discuss the prognosis with cancer patients. As to support when patients experience physical or psychological symptoms, other studies also reported a need for improvement [18, 29], although it should be stressed that full control of common symptoms like fatigue, shortness of breath and pain may not always be possible.

### Implications

Our results indicate potential room for improvement in the support provided by GPs and home care nurses to patients experiencing symptoms, especially fatigue. This could be done by giving more attention to possible symptoms, even if this means acknowledging that full symptom control may not be achievable, particularly in the final phase of the disease. Our results also indicate that it is important that information about the expected course of the illness is tailored to the individual preferences of a patient, as preferences may differ between patients and over time. However, we acknowledge that providing information about the expected course of the illness may be difficult, as this course may not always be predictable. Besides, information provision should be broader than just information about the expected course of the disease. Relating to advance care planning, it should for instance also include the opportunities for good care if a patient suffers from certain symptoms, as well as discussing the patient’s perspective on end-of-life decisions about (potentially) life-prolonging or life-shortening treatments.

## Conclusions

In conclusion, we found that most patients with advanced cancer receive the care that they consider important. Aspects that could possibly be improved are the support the GPs give when a patient experiences fatigue and discuss with patients whether they want to receive information about the expected course of the illness.

## Abbreviations

GP: General practitioner
CQ Index: Consumer quality index
COPD: Chronic obstructive pulmonary disease
